# Bridging Gaps: A Quality Improvement Project for the Continuing Medical Education on Stick (CMES) Program

**DOI:** 10.7759/cureus.62657

**Published:** 2024-06-18

**Authors:** Jessica Pelletier, Yan Li, Emily Cloessner, Vera Sistenich, Nicholas Maxwell, Manoj Thomas, Deb Stoner, Bethel Mwenze, Angellar Manguvo

**Affiliations:** 1 Emergency Medicine, Washington University School of Medicine, St. Louis, USA; 2 Center for Information Systems and Technology, Claremont Graduate University, Claremont, USA; 3 Emergency Medicine, St. George Hospital, Sydney, AUS; 4 Business Management, University of Sydney, Darlington, AUS; 5 Emergency Medicine, Evangelical Community Hospital, Lewisburg, USA; 6 Emergency Medical Services, Samaritan Health Systems, Kampala, UGA; 7 Department of Graduate Health Professions in Medicine, University of Missouri-Kansas City School of Medicine, Kansas City, USA

**Keywords:** information and communication technology, open-access medical education, low- and middle-income country (lmic), low-resource setting, low-income resource-limited countries, medical education technologies, continuous medical education, medical education curriculum, emergency medicine barriers, continuing medical education/graduate medical education/undergraduate medical education

## Abstract

Background: Aimed at bridging the gap in continuing medical education (CME) resource availability in low- and middle-income countries (LMICs), the "Continuing Medical Education on Stick" (CMES) program introduces two technological solutions: a universal serial bus (USB) drive and the CMES-Pi computer facilitating access to monthly updated CME content without data cost. Feedback from users suggests a lack of content on tropical infectious diseases (IDs) and content from a Western perspective, which may be less relevant in LMIC settings.

Methods: This quality improvement project was intended to identify areas for improvement of the CMES database to better meet the educational needs of users. We compared the CMES content with the American Board of Emergency Medicine (ABEM) Exam content outline to identify gaps. The curriculum map of the CMES library, encompassing content from 2019 to 2024, was reviewed. An anonymous survey was conducted among 47 global users to gather feedback on unmet educational needs and suggestions for content improvements. All healthcare workers who were members of the CMES WhatsApp group were eligible to participate in the survey.

Results: The curriculum map included 2,572 items categorized into 23 areas. The comparison with the ABEM outline identified gaps in several clinical areas, including procedures, traumatic disorders, and geriatrics, which were represented -5%, -5%, and -4% in the CMES library compared with the ABEM outline, respectively. Free responses from users highlighted a lack of content on practical skills, such as electrocardiogram (ECG) interpretation and management of tropical diseases. Respondents identified emergency medical services (EMS)/prehospital care (81%), diagnostic imaging (62%), and toxicology/pharmacology (40%) as the most beneficial areas for clinical practice. In response to feedback from users, new content was added to the CMES platform on the management of sickle cell disease and dermatologic conditions in darkly pigmented skin. Furthermore, a targeted podcast series called “ID for Users of the CMES Program (ID4U)” has been launched, focusing on tropical and locally relevant ID, with episodes now being integrated into the CMES platform.

Conclusions: The project pinpointed critical gaps in emergency medicine (EM) content pertinent to LMICs and led to targeted enhancements in the CMES library. Ongoing updates will focus on including more prehospital medicine, diagnostic imaging, and toxicology content. Further engagement with users and education on utilizing the CMES platform will be implemented to maximize its educational impact. Future adaptations will consider local relevance over the ABEM curriculum to better serve the diverse needs of global users.

## Introduction

Scholarly discourse has consistently highlighted significant disparities in providing continuing medical education (CME) in low- and middle-income countries (LMICs) [1,2]. These disparities are driven by multiple barriers, including limited access to context-relevant CME content from conferences, textbooks, journals, and online platforms. In addition, the lack of mandated CME standards, coupled with financial and technical challenges, further complicates the accessibility and effectiveness of CME in these regions [3,4].

In response to these issues, Techies Without Borders (TWB) [1] has developed the "Continuing Medical Education on Stick" (CMES) initiative, aimed at bridging the gap in CME resource availability in LMICs. This initiative introduces two technological solutions. The first is a universal serial bus (USB) drive that autonomously updates with new CME content via a cloud-based server when an Internet connection is available. This device is particularly beneficial in rural healthcare settings with few healthcare workers. The second solution, the CMES-Pi, is a cost-effective, Raspberry Pi-based offline computer that facilitates access to CME content on both iOS and Android devices via TWB’s custom phone application. This application enables users to download content directly to their mobile devices without incurring data costs, catering especially to low-resource environments where multiple users share a single device [5].

The content on the CMES platform includes podcasts and written summaries from numerous renowned free, open-access medical education (FOAMed) sources. The following donors have generously provided content for the CMES platform: Emergency Medicine Reviews and Perspectives (EM:RAP), Emergency Medicine Cases, emDocs.net, Don’t Forget the Bubbles, the World Health Organization (WHO), Alfred Health, MCHD Paramedic Podcast, Dr. Smith’s ECG Blog, and Life in the Fast Lane (these last two sources are pending upload) [6]. The available CME content primarily focuses on emergency medicine (EM) but is also applicable across various specialties. Moreover, local CME materials can be uploaded to a local folder on each CMES-Pi device by site administrators [5].

In 2023, TWB established new partnerships with sites in the Gambia, Nigeria, and Uganda. In October 2023, the TWB team visited the Gambia and Uganda to conduct new installations of the CMES program and held meetings with longstanding partner sites in Uganda [6]. During these installations, informal interviews were conducted with users to assess their needs. TWB is also conducting a qualitative research project designed to tailor the CMES program to the specific needs of users at four African partner sites. Feedback obtained during installations and via informal and formal interviews suggested that the CMES library lacks content on locally relevant infectious diseases (IDs), especially pertinent in rural areas [7]. This needs assessment highlighted the priority of tailoring the CMES content library more closely to meet the needs of the clinicians served [7].

In response to these findings, TWB has taken several steps. These included collating new content from FOAMed resources; initiating a monthly Zoom-based journal club, in which key podcasts of the month are discussed; and recruiting additional volunteer EM physicians - one to serve as the Medical Education Coordinator and the other to serve as the Clinical Content Coordinator [6]. Despite these efforts, further work is required to hone the CMES educational resource. Thus, an education quality improvement (QI) project has been launched with the following objectives: (1) develop an outline of the current content delivered through the CMES program by topic and disseminate this outline to global CMES users, (2) conduct a targeted needs assessment with global CMES users to determine what content they feel is missing and needed for their daily practice, and (3) formulate a strategic plan to address the current gaps in the CMES platform content.

## Materials and methods

Theoretical frameworks

**Figure 1:**
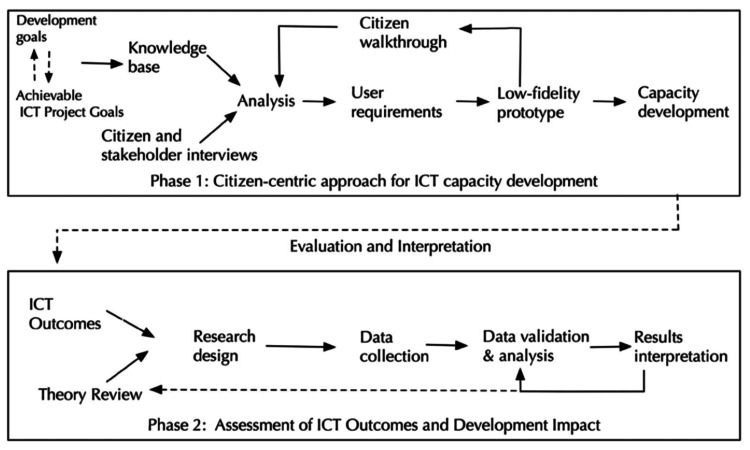
Citizen-centric approach for ICT capacity development. Reproduced with permission from Li et al. [5].

Two theoretical frameworks serve as the foundation for this educational QI project. The first framework, Citizen-Centric Capacity Development (CCD), posits that people in LMICs can advance their own information and communication technology (ICT) capacities if provided with appropriate tools [5]. Phase 1 of CCD within the CMES program, which involved implementing the CMES program in LMICs, has been completed. This educational QI project now focuses on Phase 2 of CCD (see Figure 1), which is conceptualized as a continuous cycle or process. This implies ongoing educational QI evaluations are necessary after implementing the recommendations outlined in this manuscript [5].

**Figure 2:**
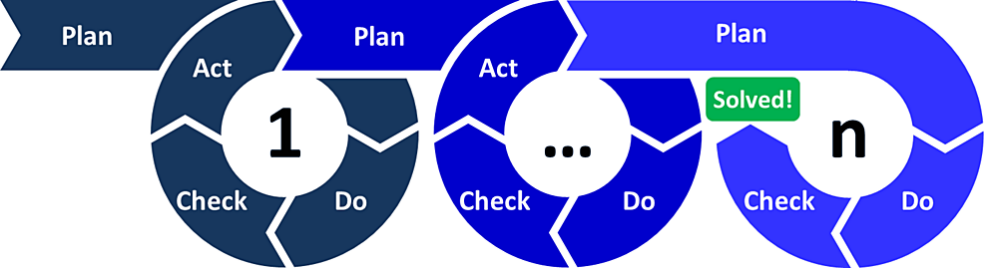
The Plan-Do-Check-Act cycle. Open-access image credit: Christoph Roser at AllAboutLean.com (CC BY-SA 4.0, https://commons.wikimedia.org/w/index.php?curid=47640479).

The second framework that underpins this project is the Plan-Do-Study-Act cycle, also known as the Plan-Do-Check-Act cycle, a methodology for continuous quality improvement [8]. Currently, this project is in the “Study” phase, which will be followed by the “Act” phase, wherein the proposed changes will be ideally implemented, leading to the commencement of another cycle (Figure 2) [8].

Curriculum map

A spreadsheet was created to categorize content similar to the topics found on the American Board of Emergency Medicine (ABEM) Exam [9]. These topics are listed in Table 1. The ABEM Exam was selected as a model due to its comprehensive coverage of content essential for emergency physicians in the United States, one of the first countries to recognize EM as a specialty [10]. Currently, nearly half of the world’s emergency physicians practice in the United States [10], making it a benchmark for EM training globally.

**Table 1:** ABEM Exam content topics comprised in the qualifying specialist emergency physician exam compared with CMES library topics selected for categorization. ABEM = American Board of Emergency Medicine, CMES = Continuing Medical Education on Stick, EMS = emergency medical services, GI = gastrointestinal, HEENT = head, ears, eyes, nose, and throat

ABEM Exam		CMES library
Medical knowledge, patient care, and Procedural Skills	Abdominal and GI	Abdominal and GI
	Cardiovascular disorders	Cardiovascular disorders
	Cutaneous disorders	Cutaneous disorders
	N/A	EMS
	Endocrine, metabolic, and nutritional disorders	Endocrine/metabolic/nutritional
	Environmental disorders	Environmental/wilderness medicine
	N/A	Evidence-based medicine
	HEENT disorders	HEENT
	Hematologic disorders	Hematologic/oncologic
	N/A	Imaging
	Systemic infectious disorders, immune system disorders	Immune system/infectious
	Musculoskeletal disorders (non-traumatic)	Musculoskeletal (non-traumatic)
	Nervous system disorders	Nervous system/neurology
	Obstetrics and gynecology	Obstetrics and gynecology
	Renal and urogenital disorders	Renal and urogenital
	Procedures and skills	Procedures and skills, including ultrasound
	Psychobehavioral disorders	Psychosocial/social determinants of health
	Signs, symptoms, and presentations	N/A
	Thoracic-respiratory disorders	Thoracic/respiratory, including airway
	Toxicologic disorders	Pharmacology/toxicology
	Traumatic disorders	Traumatic disorders
	Other components	Unknown/other
Physician Tasks	Geriatrics	Geriatrics
	Pediatrics	Pediatrics

In developing the curriculum map, specific consideration was given to the searchability of topic tags, such as “hematology” and “cardiology.” The categories, such as “signs, symptoms, and presentations,” were considered to be too general and were not included. Overlapping content led to the consolidation of certain categories: “immune system disorders” with “systemic infectious diseases” and “psychobehavioral disorders” with content related to social determinants of health into “psychosocial.” “Ultrasound” was categorized under “procedures and skills.” The final list of content areas in the CMES curriculum map is detailed in Table 1.

For each item added to the curriculum map from the CMES content library, characteristics, such as content provider, year, month, PDF name, podcast name, current tags, and added tags, were recorded. Upon completion, the representation of each content category within the CMES library was quantified by dividing the number of items in each category by the total number of items in the library. The value was then compared to the corresponding topic coverage on the ABEM Exam to identify any discrepancies.

CMES Curriculum Redesign Survey

A Curriculum Redesign Survey was developed by the authors of the QI project. The institutional review board (IRB) at Washington University in St. Louis waived the need for IRB review of this project given that the survey study was anonymous and for QI purposes only. The survey questions, detailed in Table 2, were formulated collaboratively by the project team. Response options for the first question were determined through consensus among the TWB team, which consists of 17 information technology and healthcare volunteers from nine countries: Australia, China, Germany, India, Nepal, Romania, Taiwan, Uganda, and the United States. To facilitate broad participation, a read-only version of the curriculum map and the Curriculum Redesign Survey were shared with the CMES Global Community group on WhatsApp, the primary communication channel for the TWB team to make announcements to CMES users. Users from all 19 countries in which the CMES program is installed have access to the WhatsApp group. The Curriculum Redesign Survey was created in Google Forms, and responses were anonymous. No demographic data were collected to preserve anonymity. Survey responses were collected between April 8, 2024 and April 25, 2024, and a total of four reminder messages were disseminated via the WhatsApp group after the initial survey announcement was released.

**Table 2:** CMES Curriculum Redesign Survey questions. These topics were selected based on the curriculum map partway through completion, at which time these topic categories were noted to be the most deficient in the CMES library compared with the ABEM Exam. EMT = emergency medical technician, CNA = certified nursing assistant, LPN = licensed practical nurse, RN = registered nurse, NP = nurse practitioner, ID = infectious disease

Question number	Question	Response options
1	What is/are your role(s) in healthcare?	Physician - attending/consultant
		Physician - resident
		Physician - house officer
		Medical student
		Pharmacist
		EMT/paramedic
		Nurse - CNA/LPN
		Nurse - RN
		Nurse - NP
		Nurse - advanced nurse practitioner
		Other
2	Please list any medical topics that you have searched for on the CMES-Pi or USB and have been unable to find.	Free response.
3	Which of the following has been the most challenging for you when searching for information on the CMES-Pi or USB? You may select more than one option.	British vs. American spelling
		Tags are spelled wrong
		You are missing the topics I am interested in
		The search bar doesn't allow me to search with phrases/sentences
		N/A, I haven’t searched for anything
		Other
4	Which of the following topics would be most useful for your practice? You may select more than one option.**	Emergency medical services/prehospital care
		Hematology/oncology
		Diagnostic imaging
		Psychosocial/social determinants of health
		Toxicology/pharmacology
		Other
5	Is the existing ID content on the CMES platform fulfilling your requirements? If not, kindly specify any topics that you require but are presently unavailable.	Free response

## Results

Curriculum map

A total of 2,572 content items from the CMES library, including PDF and MP3 files from the last five years (2019-2024), were cataloged into the curriculum map across 23 categories. Items were categorized under multiple categories when topics overlapped. Table 3 displays the percentage distribution of topics in the ABEM Exam and the CMES library, along with the difference between them. A comparison of the CMES library content with the ABEM Exam Content outline revealed that the percentage of available CMES content was lower than that of the ABEM in several categories, particularly procedures and skills (-5%), traumatic disorders (-5%), geriatrics (-4%), gastrointestinal (GI) (-3%), and thoracic/respiratory disorders (-3%). The only domain in which the CMES library greatly exceeded the ABEM percentage was unknown/other (+5%).

**Table 3:** Percentages by topic on the ABEM Exam compared with the CMES library. ABEM = Continuing Medical Education on Stick, CMES = Continuing Medical Education on Stick, GI = gastrointestinal, EMS = emergency medical services, HEENT = head, ears, eyes, nose, throat

Topic	ABEM Exam %	CMES library %	Difference
Abdominal and GI	0.07	0.04	-0.03
Cardiovascular disorders	0.10	0.09	-0.01
Cutaneous disorders	0.03	0.02	-0.01
EMS	0.00	0.03	+0.03
Endocrine/metabolic/nutritional	0.05	0.04	-0.01
Environmental/wilderness medicine	0.02	0.01	-0.01
Evidence-based medicine	0.00	0.02	+0.02
Geriatrics	0.06	0.02	-0.04
HEENT	0.04	0.02	-0.02
Hematologic/oncologic	0.03	0.04	+0.01
Imaging	0.00	0.01	+0.01
Immune system/infectious	0.09	0.09	0.00
Musculoskeletal (non-traumatic)	0.03	0.03	0.00
Nervous system/neurology	0.06	0.05	-0.01
Obstetrics and gynecology	0.03	0.04	+0.01
Pediatrics	0.08	0.07	-0.01
Procedures and skills, including ultrasound	0.08	0.03	-0.05
Psychosocial/social determinants of health	0.02	0.04	+0.02
Renal and urogenital	0.03	0.02	-0.01
Thoracic/respiratory, including airway	0.07	0.04	-0.03
Pharmacology/toxicology	0.04	0.15	+0.11
Traumatic disorders	0.09	0.04	-0.05
Unknown/other	0.02	0.07	+0.05

Curriculum Redesign Survey 

A total of 348 CMES participants received a read-only copy of the curriculum map and received requests to complete the CMES Curriculum Redesign Survey via the CMES Global Community group on WhatsApp. Of these, 47 individuals completed the survey, resulting in a response rate of 14%.

**Figure 3:**
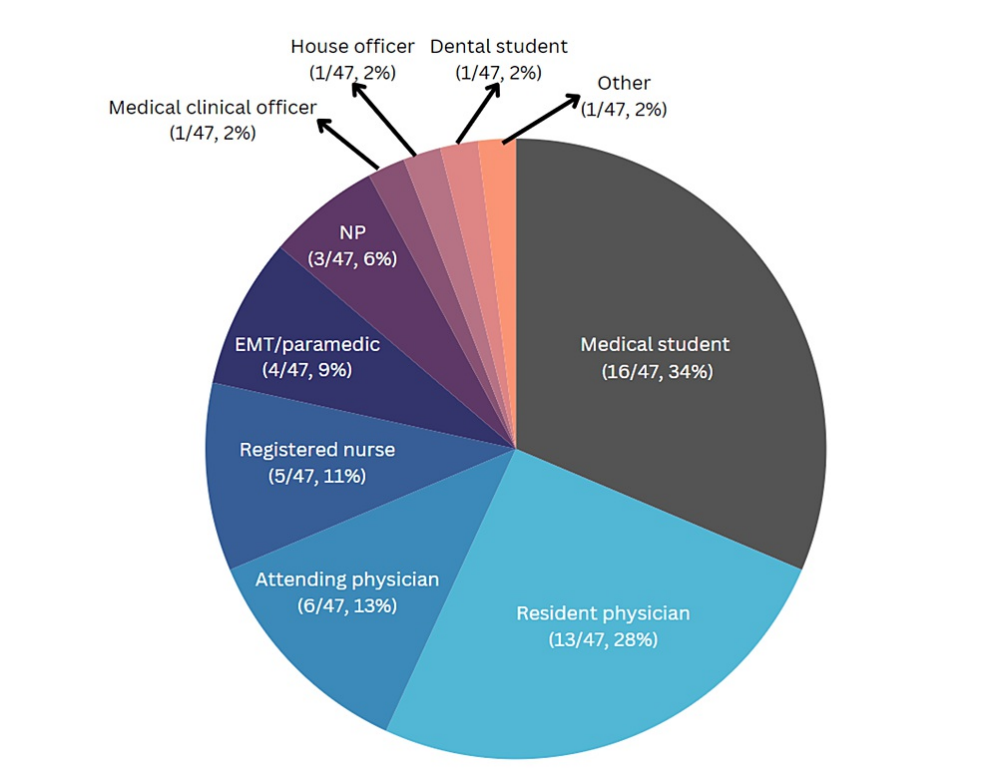
Breakdown of survey respondents by healthcare role. Numbers of respondents and percentages are indicated in parentheses. EMT = emergency medical technician, NP = nurse practitioner

In response to the first question, “What is/are your role(s) in healthcare,” the largest group of survey respondents were medical students (34%), followed by resident physicians (28%), attending/consultant physicians (13%), registered nurses (11%), prehospital personnel (9%), nurse practitioners (6%), house officers (2%), medical clinical officers (2%), dental students (2%), and other healthcare workers (2%) (Figure 3).

Regarding the second question, “Please list any medical topics that you have searched for on the CMES-Pi or USB and have been unable to find,” the respondents identified several topics not found or sparsely available on the server. These included guidelines on the management of sickle cell disease, the management of chronic musculoskeletal pain, prehospital airway management, disaster medicine, electrocardiography (ECG), and preclinical content (examples suggested by survey respondents included cardiovascular development and development of the urinary system). In addition, topics like hydrocephalus, atrial fibrillation, rapid sequence intubation, and medical ethics were cited as missing, although they were, in fact, present in the library.

**Figure 4:**
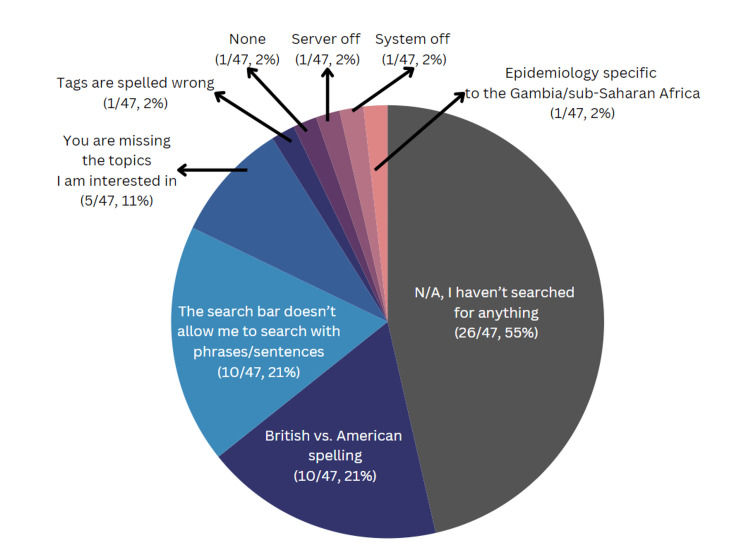
Challenges encountered by users when searching for information on the CMES platform. Numbers of respondents and percentages are indicated in parentheses. CMES = Continuing Medical Education on Stick, N/A = not applicable

In response to the third question, “Which of the following has been the most challenging for you when searching for information on the CMES-Pi or USB,” 55% of the respondents reported that they had not searched for anything on the CMES platform (Figure 4). The specific topics previously mentioned as missing we specifically cited as gaps in the server’s content.

For the fourth question, “Which of the following topics would be most useful for your practice,” the topic areas cited as being most useful for clinical practice included EMS/prehospital care (81%), diagnostic imaging (62%), toxicology/pharmacology (40%), hematology/oncology (28%), and psychosocial/social determinants of health (28%). In addition, suggestions from users included preclinical content (physiology/anatomy) (2%), pediatric emergencies (2%), nephrology (2%), psychiatric medications (2%), and other (4%).

For the fifth question, “Is the existing ID content on the CMES platform fulfilling your requirements,” elicited responses highlighted the need for more comprehensive coverage on dermatological conditions in patients with black-colored skin (one user), malaria (one user), tuberculosis (one user), neglected tropical diseases (one user), common ID as relevant to each local country (one user), and antimicrobial resistance (one user). One individual noted, “I very rarely need it so I have not searched or used it,” leaving it unclear whether this was a reference to the ID content or the CMES program in general.

## Discussion

This educational QI initiative was launched to refine the content of the CMES platform to better align with user preferences [11]. In low-resource settings, low doctor-to-patient ratios, and limited resources make it challenging for clinicians to meet patient needs [11]. CMES serves as a clinical decision support tool (CDST) designed for bedside clinical teaching and continuous knowledge improvement at home. Information and communication technologies for development (ICT4D) like CMES are theorized to be very useful for making progress in developing communities [12]. While CDSTs are beneficial in resource-rich settings, many are not suitable in LMICs due to the limited availability of the Internet and electricity. This exacerbates health disparities between resource-rich and resource-poor settings [13]. Efforts such as texting-based CDSTs [14] have been initiated to overcome these challenges, the CMES program stands out as a unique ICT4D solution for lower-resource healthcare settings because it works without the need for constant internet and electricity access [15]. 

Medical students were the largest respondent group in the survey (34%) (question 1), raising questions about whether this reflects a response bias or if they are the most active CMES users. To assess whether medical students are more engaged than other user groups, a comprehensive survey of user roles across all participating sites is necessary. This could reveal whether the CMES program needs to adjust its content to include more preclinical materials specifically designed for medical students, representing a significant shift from the program’s original focus. Introducing a culture of ongoing learning early in medical education could potentially increase the future use of CME resources [16]. The CMES platform could be particularly useful for medical students during their early learning phases, as studies have shown a preference for e-learning resources over traditional educational materials like books or lecture handouts among modern medical students. 

One objective of the CMES program is to foster a culture of continuous education at participating sites. Where such a culture already exists, CMES seeks to strengthen and enrich it [17]. It is suggested that enhancing the quality of care in resource-constrained settings should start with improving the educational experiences of medical students [18], who are the next generation of clinicians that will influence continuous learning cultures within their respective hospital systems. They also have the potential to drive quality improvement even during their undergraduate medical education [19]. By prioritizing medical students, CMES may more effectively promote CME in low-resource environments. 

It is well-documented in our CMES user database (which is not publicly available) that CMES has a higher number of medical student users at a particular partner site in the Gambia compared with all other global CMES partner sites with 17 medical students from the Gambia, far outnumbering those from Uganda and Nepal. Although the Curriculum Redesign survey did not collect data on the respondents' locations, it is likely that a larger number of responses came from Gambian medical students. This may indicate a local context bias, suggesting different challenges that make them more reliant on CMES. Future surveys should collect data on respondents’ locations to determine if curriculum changes should be site-specific or global.

The results underscore the need to address content deficiencies on the CMES platform. For example, the integration of the "Occlusion Myocardial Infarction (OMI) Manifesto" from Dr. Smith's ECG blog into CMES, despite formatting challenges, shows efforts to meet specific user needs. Moreover, the majority of survey respondents (55%) reported they had never searched the CMES library. Possible reasons for this include the availability of alternative CME resources, inadequate training on how to use the platform, or logistical barriers such as limited access to devices. Additional barriers, such as the language barrier since CMES content is only available in English, and the limited relevance of content to local contexts, also contribute to this disengagement. Addressing these issues requires understanding the specific barriers faced at each site, which could be achieved through qualitative interviews with CMES users. The TWB team could also deploy the “Assessing The Learning Strategies of Adults” (ATLAS) survey to better understand how adult learners prefer to integrate a new learning activity into their routine, which could further help tailor the CMES content to better fit user preferences [20].

Furthermore, only one respondent mentioned issues with inappropriate tagging (question 3), which complicates the search process. Although many tags were corrected, ongoing encryption issues have hampered complete resolution, indicating a need for ongoing oversight by a clinician volunteer. In addition, 21% of users reported difficulties with the search function of the CMES devices, primarily because it only accepts words or phrases, not complete sentences or questions. This functionality is less intuitive compared to more familiar search tools like Google or artificial intelligence (AI) systems such as ChatGPT, leading to potential user frustration. Research suggests that generative AI may help improve research equity and personalized learning [21,22]. Future improvements could include enhancing the search function with generative AI capabilities, which would eliminate the need for manual tagging and improve search accuracy and usability. 

To address the issue of spelling variations between British and American English spelling reported by some users (21%) (question 3), efforts have been made to include both British and American English spellings to enhance searchability. In addition, integrating generative AI into the CMES search function would allow it to recognize misspelled words and the variations between British and American. This enhancement would make CMES more accessible to a broader range of English, including those who are not native speakers [23].

Some free-response answers to question 3 mentioned content issues (i.e., lack of epidemiology specific to sub-Saharan Africa), although the question was intended to elicit data regarding process issues when using the CMES platform. Future CMES surveys should specify that "Other" refers to comments on process issues to ensure that this is clear to survey respondents.

There is a significant demand for more prehospital content, as indicated by 81% of survey respondents (question 4). This is surprising, given that previous studies in the United States have demonstrated a low uptake of FOAMed resources among prehospital providers in some regions [24]. A recent study in South Africa showed similar FOAMed usage among emergency physicians to their counterparts in high-income settings [25]. One study to date has demonstrated the usability of FOAMed for EMS providers in a middle-income setting [26]. Data on FOAMed uptake by prehospital providers in low-resource settings is still lacking. It is plausible that the landscape of FOAMed by EMS differs in resource-constrained settings. This warrants further investigation. While some content is provided by sources such as EM:RAP, emDocs.net, and the MCHD Paramedic Podcast, with additional resources expected from Life in the Fast Lane, more efforts are necessary to identify additional FOAMed resources to meet this need. Similarly, diagnostic imaging was noted by 62% of respondents as a content domain crucial to their work (question 4), a domain where previous attempts to solicit donations were unsuccessful. Thus, the CMES team must explore additional diagnostic imaging FOAMed resources.

Interestingly, although the CMES library is well-stocked with ID content compared to the ABEM Exam standards, survey respondents reported that several ID topics were absent (question 5). This discrepancy suggests that the ID content may not fully meet the needs of low-resource settings. Prior studies have highlighted the need for open-access ID learning materials in resource-constrained regions [27], particularly given that the burden of ID is higher in these settings [28]. In light of this, efforts should be made to enrich the CMES ID content, focusing particularly on tuberculosis, malaria, tropical diseases, and locality-specific ID. This effort could involve epidemiological research at each site and direct feedback from users to identify which infectious processes are most pertinent locally.

Lastly, while users did not identify them as majorly deficient, several content domains were found to be lacking when compared with the ABEM Exam standards: procedures and skills (-5%), traumatic disorders (-5%), geriatrics (-4%), gastrointestinal (-3%), and thoracic/respiratory disorders (-3%). The lack of feedback on these deficiencies might suggest that they are not considered high-priority topics by users. The TWB team should consider conducting further surveys with CMES users to determine whether more content in these domains is truly needed, as emergency medical education must be tailored to local needs [29,30]. Addressing these content deficiencies could add more resources from sites like Emergency Medicine Cases and Life in the Fast Lane. 

A summary of recommendations for improvement of the CMES educational initiative can be found in Table 4.

**Table 4:** Recommendations for improvement of the CMES educational initiative. CEMS = Continuing Medical Education on Stick, AI = artificial intelligence, OMI = occlusion myocardial infarction, EMS = emergency medical services, ID = infectious disease, GI = gastrointestinal. *Even if connections are intermittent, they maximize the usability of the CMES phone application.

Recommendation number	Recommendation
1	Conduct another survey by site to determine whether medical students are the largest group of users If they are, consider adding preclinical content to the CMES platform
2	Upload the OMI Manifesto to the CMES platform
3	Continue qualitative interviews to determine barriers to programmatic engagement
4	Systematically encourage participating sites to keep CMES-Pi devices plugged into the internet and ethernet*
5	Identify a clinician volunteer to tag content
6	Consistently tag content with both British and American English spellings
7	Consider modifying the search feature to mimic commonly used search engines, including incorporation of generative AI
8	Collate more of the following content based on user requests: Diagnostic imaging, ECG interpretation, EMS, locally-relevant ID
9	Conduct further surveys to determine whether domains lacking compared with ABEM Exam standards are needed in local contexts

Limitations

The primary limitation of this study was the low response rate and small sample size compared with the target population of CMES users. As indicated by the best practices in survey methodology, sending more personalized invitations rather than mass messages, offering frequent reminders, providing financial incentives, and using additional recruitment strategies besides WhatsApp (such as email or social media) should be employed in the future to maximize response rates [31].

The survey was developed during the development of the curriculum map, a period marked by ongoing updates to the CMES-Pi with new materials. Thus, the content available at the beginning of our curriculum mapping did not perfectly match those available upon completion. This may have potentially biased the survey results, particularly with regard to question 4.

Moreover, response bias is a concern, as respondents might differ from non-respondents. It is plausible that the percentage of individuals with access to the CMES program who do not utilize it could exceed 55%. Those who refrain from using the CMES or CMES-Pi may also be less likely to participate in the WhatsApp group or respond to surveys. Notably, there is a CME culture at some sites in which individuals expect to receive payment (known as “facilitation”) or CME credit for participating in CME activities. Given that no financial incentive or CME credit was provided for participation in the survey, this may have unintentionally influenced who responded to the survey. 

A large number of medical students responded to this survey. If the CMES program plans to continue focusing on practicing clinicians as target users, future surveys should target only this population to clarify their needs. Furthermore, it is noteworthy that the largest group of respondents (medical students) may predominantly come from a single country. Since demographic data were not collected, it is plausible that the results reflect the biases of a particular local context as opposed to the global CMES community.

Finally, the appropriateness of using the ABEM Exam as a standard for developing EM curricula in LMICs remains uncertain, given distinct disease burdens and variations in clinic resources across these regions. Although the ABEM Exam may serve as a reasonable benchmark, it cannot be assumed to be a universally applicable model for EM education outside the United States. Future research should aim to compare the CMES content with localized EM examinations, such as the examination administered by the Fellowship of the College of Emergency Medicine of South Africa (FCEM(SA)) [32], which are tailored to meet the specific needs and conditions of the regions where TWB operates.

## Conclusions

CMES is a high-impact educational resource for healthcare workers in LMICs, providing a large number of clinicians with free CME in low-resource settings. This educational QI project has successfully identified specific areas where the CMES content library falls short of meeting the diverse needs of its users. To enhance the impact and relevance of the CMES, these deficiencies must be systematically addressed to better tailor the content to the local contexts and requirements of the targeted users.
